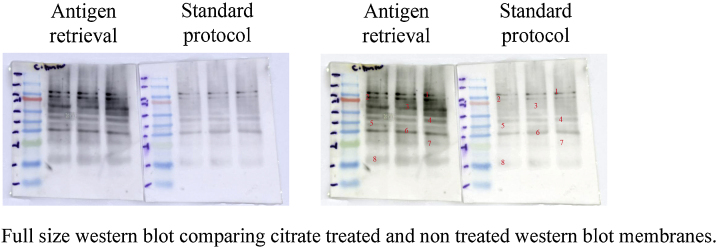# Antigen retrieval by citrate solution improves western blot signal

**DOI:** 10.1016/j.mex.2019.02.030

**Published:** 2019-02-27

**Authors:** Daniel Patiño-García, Nadia Rocha-Pérez, Ricardo D. Moreno, Renan Orellana

**Affiliations:** a Departamento de Fisiología, Facultad de Ciencias Biológicas, Pontificia Universidad Católica de Chile, Santiago, Chile; b División de Obstetricia y Ginecología, Escuela de Medicina, Pontificia Universidad Católica de Chile, Santiago, Chile; c Universidad Bernardo O’Higgins, Facultad de Salud, Departamento de Ciencias Químicas y Biológicas, Santiago, Chile

**Keywords:** Antigen Retrieval for Western Blot Technique (ARWB method), Antigen retrieval, SDS-PAGE, Citrate buffer, Antibody/epitope interaction, Novel step

## Abstract

In the present work, we describe and evaluate an additional step to the standard western blot protocol to increase signal strength after revealing. Weak or absence of signal is a common issue in western blot protocol leading to unexpected results. In our Antigen Retrieval for Western Blot Method (ARWB method), after transfer, the membrane was incubated in a citrate buffer following normal antigen retrieval procedure used for immunohistochemistry. Later, standard protocol was performed in order to reveal and compare with unexposed membranes to this antigen retrieval step. Signal in bands obtained by the modified protocol resulted significantly higher (in all 13 antibodies analyzed) compared to standard protocol. Some bands were only visible after citrate incubation. This method is a simple and economical way to improve results in western blot analysis.

• The ARWB method significantly increases band’s density in all antibodies analyzed.
• Protein localization does not influence the efficacy of the ARWB method since membrane and citoplasmatic proteins bands increase their signal in a similar way after the protocol is performed.
• This ARWB method is simple, safe, economical and undoubtedly helpful in immunoblotting for proteins with weak signal.

## Graphical abstract

**Figure:**
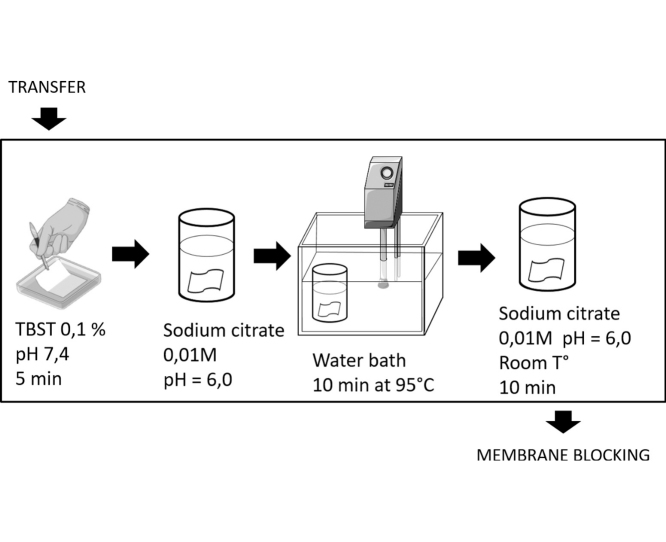

**Specifications Table**

**Table:** 

**Subject Area:**	Biochemistry, Genetics and Molecular Biology
**More specific subject area:**	Molecular Biology
**Method name:**	Antigen Retrieval for Western Blot Technique (ARWB method)
**Name and reference of original method:**	Western Blot Technique [1]
**Resource availability:**	This work was supported by FONDECYT (Grants 11170603 to R.O. and 1150352 to R.D.M)

## Method details

### Methodology background

**Fig. 1:**
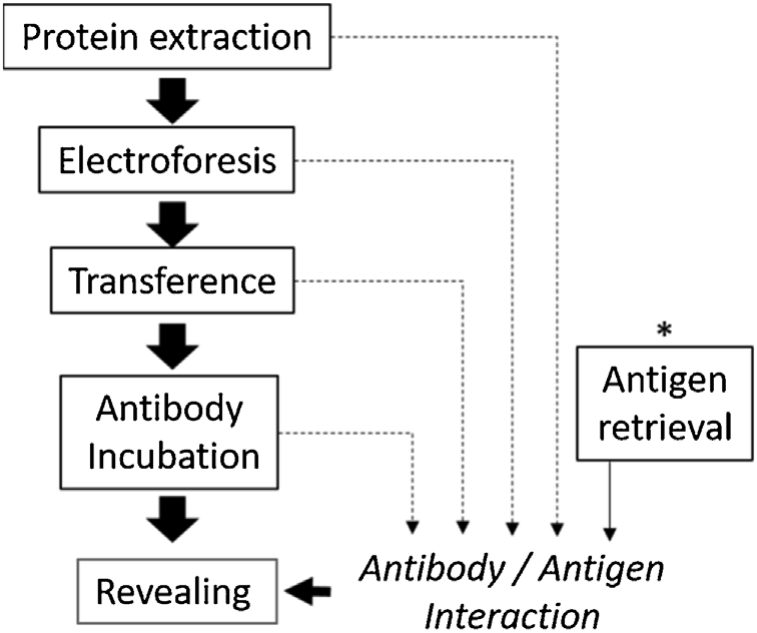
Western blot protocol diagram. Sequence on the left correspond to normal procedure. * = novel step proposed by the authors.

Western blot is one of the most-widely implemented techniques in molecular biology used to study and quantify proteins. However, even though the procedure is simple, its many steps increase the number of variables under control, making it difficult to identify and choose a specific process to modify in order to improve results (Fig. 1). Regarding the most common issues, we found: heterogeneous transfer of protein to membrane, bended migration front during electrophoresis and weak signal due to antibody or antigen [1]. Antibody binding to its specific protein target is a final step in western blot procedure and signal intensity after revealing is dependent of this interaction (Fig. 1). There are many factors affecting antibody/antigen interaction such as protein degradation, antibody availability and revealing process. Therefore, when results are inconsistent and scientists suspect that a protocol mistake arose, it is hard to isolate the step in conflict.

There are other techniques for protein analysis based on antibody-antigen interaction, which also have their own limitations and protocol issues related to result´s improvement. In immunohistochemistry for example, it is widely accepted that chemical fixation, processing and embedding media, reduce the total amount of antigen available to interact with the antibody, a problem for immunohistochemistry purposes [2,3]. For this reason, antigen retrieval step is a critical procedure in this technique [3] and it consists on incubating the samples in sodium citrate buffer on a water bath which results in exposure of antigen binding sites, thus increasing the amount of antibody/epitope interaction [[4], [5], [6], [7], [8]]. Despite this method being simple and easy to implement in a laboratory, antigen retrieval has not been incorporated in western blot technique. In this paper, we evaluate antigen retrieval impact in western blot comparing its effects respect to the standard protocol currently used.

### Sample collection and preparation

Testes of C57BL/6J mice and human endometriotic cells lines (Hs 832 and 11Z) were used for this study. Protein extraction was performed as previously published [9]. Briefly, the homogenization of both testes and cells was performed in a radio immunoprecipitation assay buffer (RIPA), with a protease inhibitor cocktail (Sigma) and a phosphatase inhibitor cocktail with 2 mM 4-(2-aminoethyl) benzenesulfonyl fluoride hydrochloride, 0.3 μM aprotinin, 130 μM bestatin hydrochloride, 14 μM E-64, 1 mM EDTA, and 1 μM leupeptinhemisulfate. Proteins were purified by centrifugation at 12,000 × *g* at 4 °C for 10 min and subsequently quantified by Bradford method.

### Western blot and antigen retrieval

For western blot analysis, 20 μg of protein were separated by electrophoresis on a 10% polyacrylamide protein gel (SDS–PAGE) under denaturing (SDS) and reducing (β-mercaptoethanol) conditions for 1.5 h and then transferred to a nitrocellulose membrane (Thermo Scientific) at 350 mA during 2 h. Then, antigen retrieval was performed as follows: nitrocellulose membrane was washed with 0.1% (v/v) Tween Tris-buffered saline solution (TBST 0.1%, pH 7.4) for 5 min at room temperature. Later, membranes were incubated with sodium citrate solution 0.01 M, pH 6.0, for 10 min at 95 °C in a water bath to expose the antigens. Next, membranes were transferred to a new recipient with sodium citrate solution at room temperature for 10 min. Finally, membranes were washed with TBST 0.1%, pH 7.4 for 5 min and blocked 1 h with a solution of 3% (w/v) BSA in TBS Tween 0,1%, and incubated overnight, at 4 °C, with a primary antibody (see Table 1). Secondary antibodies conjugated with horseradish peroxidase (KPL, Gaithersburg, MD) were incubated at 1:5000 dilution in blocking solution for 1 h at room temperature. Peroxidase activity was detected by enhanced chemiluminescence kit (PerkinElmer Inc, Waltham, MA, USA).

**Table 1:** Antibodies. Same conditions were used for standard and ARWB method.

Protein Target	Dilution Used	Manufacturer	Host	Reactivity
LHCGR	1:1000	Abbexa, Cambridge, UK	Rabbit	Human, Mouse, Rat
STAR	1:3000	Abbexa, Cambridge, UK	Rabbit	Human, Mouse
CYP19A1	1:3000	Abbexa, Cambridge, UK	Rabbit	Human, Mouse
HSD17B1	1:1000	Abbexa, Cambridge, UK	Rabbit	Human
Caspase 3	1:2000	Abbexa, Cambridge, UK	Rabbit	Human, Mouse, Rat
DIABLO	1:1000	Abbexa, Cambridge, UK	Rabbit	Human, Mouse, Rat
PGAM1	1:5000	Abbexa, Cambridge, UK	Rabbit	Human, Mouse, Rat
ESR1	1:1000	Santa Cruz Biotechnology, CA, USA	Mouse	Human, Mouse, Rat
Connexin 43	1:1000	Santa Cruz Biotechnology, CA, USA	Mouse	Human, Mouse, Rat
FAS	1:1000	Santa Cruz Biotechnology, CA, USA	Rabbit	Human, Mouse, Rat
TACE	1:1000	Santa Cruz Biotechnology, CA, USA	Rabbit	Human, Mouse, Rat
PGR	1:1000	Cell Signaling, MA, USA	Mouse	Human
β-Actin	1:5000	Sigma, MO, USA	Mouse	Human, Mouse, Rat, others

### Statistical analysis

Statistical analyses were performed using GraphPad Prism version 5.0 for Windows (GraphPad Software, San Diego, CA, USA). Differences in the means observed with unpaired *t*-test and Mann-Whitney *U* test were analyzed. Statistical significance was defined as *p* <  0.05.

### Method validation

**Fig. 2:**
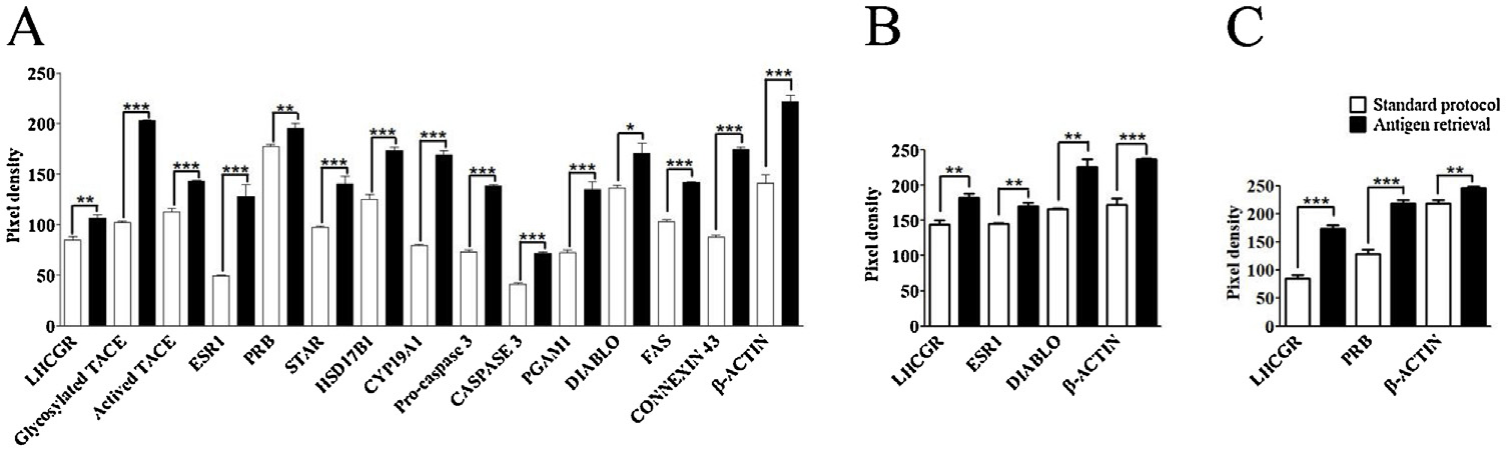
Western blot analysis obtained under standard conditions or including antigen retrieval step. A) C57BL/6J mice protein sample (N = 3). B) 11Z human endometriotic cell line (N = 3). C) Hs832 human endometriotic cell line (N = 3).

**Fig. 3:**
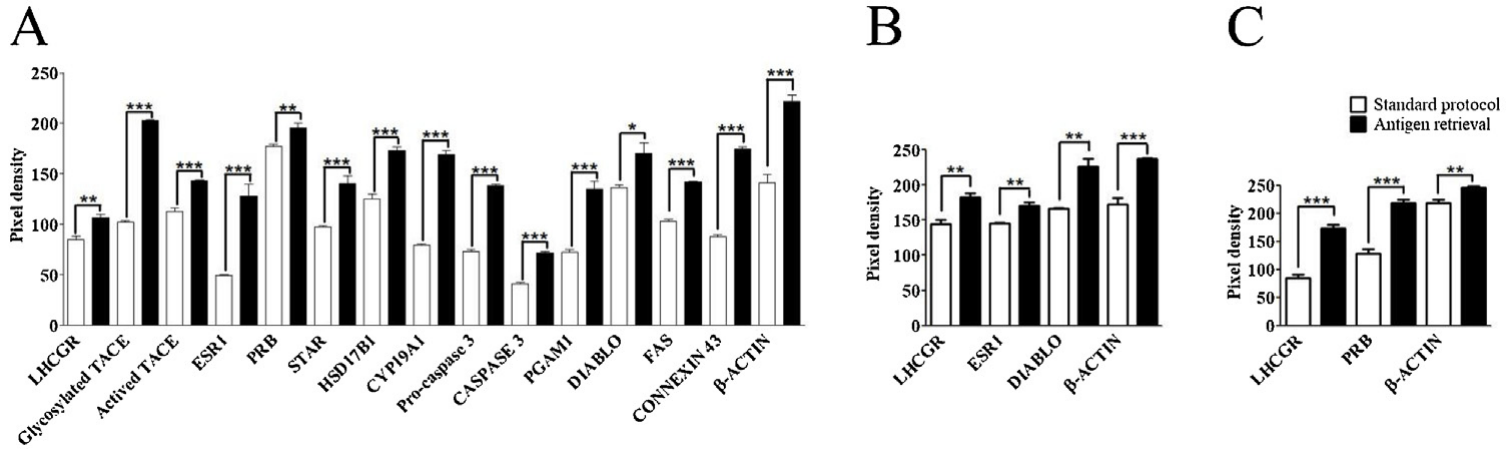
Density analysis of western blot comparing standard method and antigen retrieval step. A) C57BL/6J mice protein sample. B) 11Z human endometriotic cell line. C) Hs832 human endometriotic cell line. The mean ± standard error of the mean values are shown, Mann–Whitney *U* test, N = 3. *p < 0.05; **p < 0.01; ***p < 0.001.

In the present study, we performed western blot with 13 different antibodies (Table 1) under two different conditions, following the standard protocol or including the antigen retrieval step (Fig. 1). As we showed in Fig. 2, all antibodies presented a specific band pattern in the expected molecular weight. By comparing both protocols, the antigen retrieval step significantly increases band´s density in all antibodies (Fig. 2, Fig. 3). This result is also observed even if the antibody´s epitope and loaded protein do not belong to the same species (PRB, Fig. 2). Protein localization does not influence the efficacy of the antigen retrieval method since membrane (LHCGR, Connexin 43, TACE) and citoplasmatic proteins bands increase their signal in a similar way after the protocol is performed. Regarding other protein characteristics like molecular weight, glycosilation and disulfide bond (Supplemental Table 1), they seem do not have any effect on citrate incubation efficiency. In some proteins as TACE, ESR1, FAS and Connexin 43 the mark is strongly dependent on the citrate incubation, and it is clearly visible when this step is performed. On the other hand, for antibodies with good resolution (PRB and β-Actin) it only increases band's density. Antigen retrieval results in a global enrichment of signal of previously visible bands in western blot for all antibodies and samples analyzed in this work. No novel bands were detected by this step (Supplemental Fig. 1). For this reason, in comparative studies, control and treatment samples must be exposed to citrate incubation under the same conditions in order to exclude antigen retrieval step as a variable of interference in the results.

Regarding other methods for antigen retrieval, protease digestion was the first to be used in order to counteract the antigen masking effects of formalin fixation. However, since the advent of heat induced epitope retrieval (HIER) techniques, proteases play a much smaller role in most IHC laboratories [6]. Microwave ovens are also used for HIER, however laboratory microwaves are expensive and normal microwave ovens do not spread radiation homogenously leading to poor results. Using a pressure cooker is also an alternative, however, it is not very safe and feasible to handle as a normal water bath in a laboratory. This simple additional step is safe, economical and undoubtedly helpful in immunoblotting for proteins with weak signal. Nevertheless, more proteins should be tested in order to account it as a standard step in a western blot protocol.

## Figures and Tables

**Figure:**