# Supplementary material for: Antigen retrieval by citrate solution improves western blot signal
Source: MethodsX. 2019 Feb 27;6:464–8. doi: 10.1016/j.mex.2019.02.030 (PMC6423990; doi:10.1016/j.mex.2019.02.030)
Supplement: Supplementary file 1 [file mmc1.doc]

| **Protein** | **UniProt Entry** | **Molecular weight (kDa)** | **Glycosilation number (Positions)** | **Disulfide bond number (Position)** |
| --- | --- | --- | --- | --- |
| LHCGR | P30730  *P22888 | 100 | 7 (99, 174, 195, 291, 299, 313 and 331)  6 (103, 178, 199, 295, 303 and 317) | 1 (443↔518)  1 (439↔514) |
| STAR | P51557 | 37 | 0 | 0 |
| CYP19A1 | P28649 | 58 | 0 | 0 |
| HSD17B1 | P51656 | 35 | 0 | 0 |
| Caspase 3 | P70677 | 17 | 0 | 0 |
| DIABLO | Q9JIQ3  *Q9NR28 | 22 | 0  0 | 0  0 |
| PGAM1 | P18669 | 31 | 0 | 0 |
| ESR1 | P19785  *P03372 | 66 | 3 (10, 50 and 575)  1 (10) | 0  0 |
| Connexin 43 | P23242 | 43 | 0 | 2 (54↔192 and 187↔198) |
| FAS | P25446 | 48 | 2 (43 and 114) | 9 (44↔55, 56↔69, 59↔78, 81↔97, 100↔115, 103↔123, 125↔139, 142↔153 and 149↔165) |
| TACE | Q9Z0F8 | 120 | 7 (157, 264, 452, 498, 539, 551, and 606) | 7 (225↔333, 365↔469, 423↔453, 534↔555, 573↔582, 578↔591 and 593↔600) |
| PGR | Q00175  *P06401 | 116 | 0  0 | 0  0 |
| β-Actin | P60710  *P60709 | 40 | 0  0 | 0  0 |

**Supplemental Table 1.** Molecular characteristic of proteins used for western blot analysis

***** Human protein. Data were taken from https://www.uniprot.org/
